# A multi-scale gated multi-head attention depthwise separable CNN model for recognizing COVID-19

**DOI:** 10.1038/s41598-021-97428-8

**Published:** 2021-09-10

**Authors:** Geng Hong, Xiaoyan Chen, Jianyong Chen, Miao Zhang, Yumeng Ren, Xinyu Zhang

**Affiliations:** Department of Electrical Information and Automation, Tianjin University of Science and Technology, Tianjin, 300222 China

**Keywords:** Image processing, Radiography, Computed tomography, Computer science, Computational models, Machine learning

## Abstract

Coronavirus 2019 (COVID-19) is a new acute respiratory disease that has spread rapidly throughout the world. In this paper, a lightweight convolutional neural network (CNN) model named multi-scale gated multi-head attention depthwise separable CNN (MGMADS-CNN) is proposed, which is based on attention mechanism and depthwise separable convolution. A multi-scale gated multi-head attention mechanism is designed to extract effective feature information from the COVID-19 X-ray and CT images for classification. Moreover, the depthwise separable convolution layers are adopted as MGMADS-CNN’s backbone to reduce the model size and parameters. The LeNet-5, AlexNet, GoogLeNet, ResNet, VGGNet-16, and three MGMADS-CNN models are trained, validated and tested with tenfold cross-validation on X-ray and CT images. The results show that MGMADS-CNN with three attention layers (MGMADS-3) has achieved accuracy of 96.75% on X-ray images and 98.25% on CT images. The specificity and sensitivity are 98.06% and 96.6% on X-ray images, and 98.17% and 98.05% on CT images. The size of MGMADS-3 model is only 43.6 M bytes. In addition, the detection speed of MGMADS-3 on X-ray images and CT images are 6.09 ms and 4.23 ms for per image, respectively. It is proved that the MGMADS-3 can detect and classify COVID-19 faster with higher accuracy and efficiency.

## Introduction

Coronavirus 2019 (COVID-19) is a persistent severe acute respiratory pandemic caused by a severe acute respiratory syndrome coronavirus 2 (SARS-CoV-2) infection. On 30 January 2020, the epidemic was declared to an international public health emergency (PHEIC) by the World Health Organization (WHO)^1^. On 11 February 2020, the novel coronavirus disease was named COVID-19, and it was recognized as a pandemic on 11 March^2^. As of 28 June 2021, more than 180 million confirmed cases of COVID-19 have been reported globally since the outbreak began, with more than 3.9 million confirmed deaths^3^.

The clinical symptoms of patients infected with COVID-19 are similar to other viral upper respiratory diseases^4^. According to clinical observations, most COVID-19 cases have common features on X-ray and CT images, such as multiple small patchy shadows, interstitial changes, and glassy turbidity^5,6^. Further symptoms developed into multiple ground-glass and infiltration shadows in both lungs. In severe cases, lung consolidation and pleural effusion may occur on the X-ray and CT images^7,8^.

Due to chest X-ray images and CT images can display the features of respiratory diseases, they become the important tools for rapid identification and early diagnosis of COVID-19^9,10^. With the continuous development of computer-aided diagnosis (CAD) technology, the detection accuracy and speed have become essential and significant for clinical application. Early in 2017, Wang et al. constructed a dataset of frontal Chest X-ray images and proposed an algorithm to diagnose 14 common respiratory diseases^11^. Rajpurkar et al. detected pneumonia from chest X-ray images with better results over the average level of radiologists^12^.

Since 2020, in order to combat COVID-19, lots of experts and researchers employed artificial intelligence technique to seek for the best or the fittest model to recognize COVID-19 accurately and efficiently from accessible X-ray and CT images.

Generally speaking, one of the classification method is to conduct a binary classification study on X-ray images, that is, COVID-19 or non-COVID-19. Hemdan et al. proposed a deep learning framework COVIDX-Net, including seven different deep convolutional neural network (CNN) models which were MobileNetV2, VGGNet-19, InceptionV3, DenseNet-201, ResNetV2 InceptionResNetV2, and Xception. The COVIDX-Net network was performed a binary classification experiment on 50 chest X-ray images, and the accuracy of classification was up to 91%^13^. Abbas et al. performed a binary classification experiment on COVID-19 X-ray images by a deep CNN DeTraC to optimize the backbone network achieving accuracy of 95.12%^14^. However, the experimental dataset was small and unbalanced.

The other classification method is the multiclass classification on X-ray images that most experts and researchers pay more attention to. Ozturk et al. proposed a deep CNN model DarkCovidNet on chest X-ray images with the accuracy of 87.02% for three-classification (COVID-19, No-Findings and Pneumonia)^15^. Khan et al. proposed a deep CNN model CoroNet based on the Xception architecture, which was pre-trained on the ImageNet dataset and was performed four-classification (normal, pneumonia bacterial, pneumonia viral and COVID-19) experiments on 1300 X-ray images with an overall accuracy of 89.6%^16^. Wang et al. introduced a deep CNN model COVID-Net, and performed three-classification experiments (COVID-19, normal and pneumonia) on 13,975 X-ray images. The overall accuracy of COVID-Net reached 92.4%. When four-classification experiments (COVID-19, viral pneumonia, bacterial pneumonia and normal) were performed, the overall accuracy reached 83.5%^17^.

Besides X-ray images, some researchers used CT images to classify COVID-19. Singh et al. designed a CNN model based on multi-objective differential evolution, which was optimized by the multi-objective fitness function. For binary classification on chest CT images, the accuracy of the model achieved 93.4%^18^. Li et al. trained a COVNet model based on the ResNet, and performed a binary classification experiment on 4356 chest CT images. The result showed a sensitivity of 90%^19^. Zheng et al. designed a DeCovNet model based on weak supervision method. The DeCovNet model used pre-trained UNet to segment the lung area, and feed the segmented 3D lung area into 3D deep neural network. The binary classification experiment was performed on 499 CT images. The model had an accuracy of 90.1% and a sensitivity of 90.7%^20^. Song et al. designed a Details Relation Extraction neural model (DRE-Net) to extract the top-K details in the CT images and obtain the image-level predictions. The DRE-Net model performed binary classification experiment (COVID-19 and bacterial pneumonia) on 1485 CT images. The accuracy of model achieved 94.0%^21^.

According to above literature investigations, it is clear that the accuracy of classification can continue to improve with complex structures and deeper layers. But complex structures and deeper layers will cause excessive parameters and large size of the model, which can reduce the detection speed. In practice, it is hoped to get the diagnosis result as soon as possible, so a lightweight CNN model is more practical and valuable in clinic. Inspired by the ideas of Xception^22^ and Attention^23^, this paper designs a novel lightweight CNN model using the depthwise separable convolution and attention mechanism, aiming to improve the detection accuracy, speed, and efficiency. This paper collects 17,439 X-ray images and 10,839 CT images of COVID-19 to build datasets. Through training, validation and testing, the proposed models are compared with the mainstream CNN-based models LeNet-5, AlexNet, GoogLeNet, ResNet and VGGNet-16. Furthermore, MGMADS-CNN with 1, 2, 3 attention layer are also compared.

The key techniques introducted in this article are stated briefly as follows. First, the proposed MGMADS-CNN is a novel model motivated by attention mechanism and separable convolution ideas. The model structure is specified in details in the article, which is verified as a satisfied classifier superior to the compared models after testing. Second, the MGMADS-CNN model has a smaller size by reducing the quantity of calculation parameters, which is easy to transplant and deploy in clinic terminal devices. Third, the data enhancement and expansion techniques are adopted to solve the unbalance problem of datasets, which avoid overfitting and increase the generalization ability of the model.

## Methodology

### MGMADS-CNN model

Aiming to progress the detection accuracy and reduce the model size, a novel CNN-based model inspired by the multi-scaled gated multi-head attention mechanism, namely MGMADS-CNN, is proposed.

**Figure 1:**
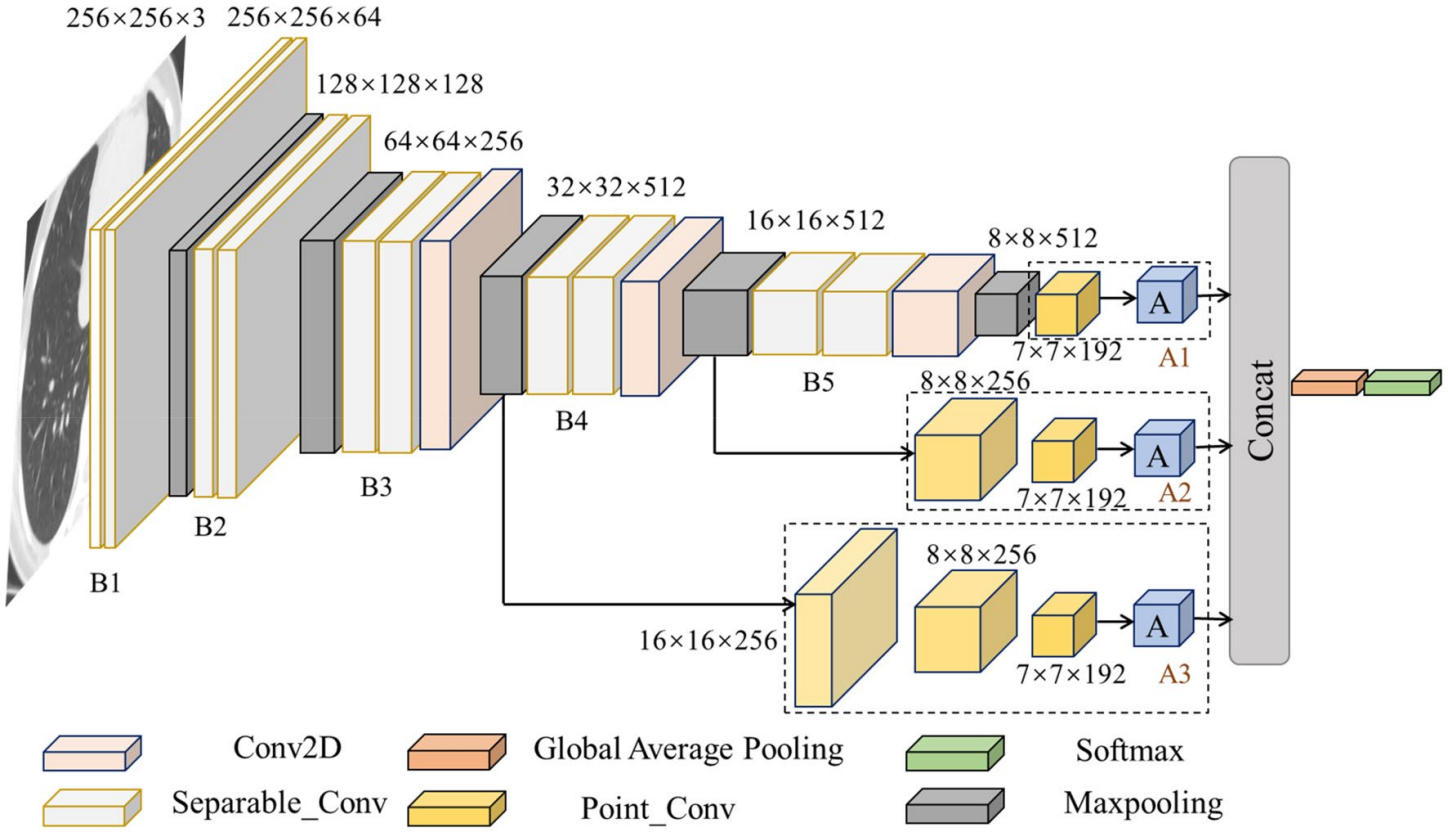
MGMADS-3 model for COVID-19 detection.

The structure of proposed MGMADS-CNN model is shown in Fig. 1, and the outputs and parameters of each layer are shown in Table 1. The model consists of convolution extraction blocks and attention modules. From left to right in Fig. 1, the first two convolutional blocks (B1 and B2 in Table 1) are composed of two depthwise separable convolution layers and a maximum pooling layer. The following three convolutional blocks (B3, B4 and B5 in Table 1) includes two depthwise separable convolution layers, a standard convolution layer, and a maximum pooling layer. Each depthwise separable convolution layer is output after batch normalization. The multi-head attention blocks represented by an ‘A’ in Fig. 1. The multiscale means the MGMADS-CNN can be feasible with different attention blocks. The MGMADS-1 means the structure including ‘A1’ in Table 1, the MGMADS-2 means the structure including ‘A1’ and ‘A2’, and the MGMADS-3 means the structure including ‘A1’, ‘A2’ and ‘A3’ in Table 1. To ensure the integrity of input information, the model uses the global average pooling instead of the fully connected layer. The global average pooling can avoid the loss of shallow feature information, and reduce the number of parameters, and prevent overfitting during the training.

**Table 1:** Layer outputs and parameters of MGMADS.

Blks	Layer (type)	Output shape	Paras	Blks	Layer (type)	Output shape	Paras
	Input layer	(None, 256, 256, 3)	0	B5	Separable_Conv2D_16	(None, 16, 16, 512)	133,888
B1	Separable_Conv2D_1	(None, 256, 256, 64)	283		Separable_Conv2D_17	(None, 16, 16, 512)	267,264
	Separable_Conv2D_2	(None, 256, 256, 64)	4736		Conv2D_18	(None, 16, 16, 512)	2,359,808
	Maxpooling2d_1	(None, 128, 128, 64)	0		Maxpooling2d_5	(None, 8, 8, 512)	0
B2	Separable_Conv2D_3	(None, 128, 128, 128)	8896	A1	Point_Conv2D_19	(None, 7, 7, 192)	393,408
	Separable_Conv2D_4	(None, 128, 128, 128)	17,664		Multi-head attention_A_1	(None, 49, 32)	117,344
	Maxpooling2d_2	(None, 64, 64, 128)	0	A2	Point_Conv2D_14	(None, 8, 8, 256)	131,328
B3	Separable_Conv2D_5	(None, 64, 64, 256)	34,176		Point_Conv2D_15	(None, 7, 7, 192)	196,800
	Separable_Conv2D_6	(None, 64, 64, 256)	68,096		Multi-head attention_A_2	(None, 49, 32)	117,344
	Conv2D_7	(None, 64, 64, 256)	590,080	A3	Point_Conv2D_8	(None, 16, 16, 256)	65,792
	Maxpooling2d_3	(None, 32, 32, 256)	0		Point_Conv2D_9	(None, 8, 8, 256)	65,792
B4	Separable_Conv2D_11	(None, 32, 32, 512)	133,888		Point_Conv2D_10	(None, 7, 7, 192)	196,800
	Separable_Conv2D_12	(None, 32, 32, 512)	267,264		Multi-head attention_A_3	(None, 49, 32)	117,344
	Conv2D_13	(None, 32, 32, 512)	2,359,808		NormL_(123)	(None, 7, 7, 32)	64
	Maxpooling2d2D_4	(None, 16, 16, 512)	0		Layers_Add	(None, 7, 7, 32)	0
	Global_average_pooling2d_1	(None, 32)	0		dense_13 (Dense)	(None, 4)	132

The input images are 256 × 256. During the convolution sampling, it’s necessary to add image channels and the convolution kernels for reserving the image features and avoiding the information loss. After convolution, the image channels will be increased from 3 to 64, and then gradually to 512. The step size of the maximum pooling layer is 2, so the image size is one half after each maximum pooling, the scale of the image is gradually reduced and the image information is continuously compressed. The ‘(None, 256, 256, 3)’ in Table 1 means the output shape of each layer in the model. ‘None’ means that the ‘batch_size’ is not specified. ‘(256, 256)’ is the size of the input feature map. ‘3’ is the number of channels in the feature map.

The loss function of MGMADS-CNN is categorical_crossentropy loss function, which is used to evaluate the difference between the probability distribution obtained by the training and the real ones. It describes the distance between the actual output (probability) and the expected output (probability), that is, the smaller the value of cross entropy, the closer the two probability distributions are. The formula of the categorical_crossentropy loss function is:

1 $$C = - \frac{1}{n}\mathop \sum \limits_{x} \left[ {y\;ln\;a + \left( {1 - y} \right)ln\left( {1 - a} \right)} \right]$$

where ***C*** represents the loss function, *x* represents the sample, *y* represents the actual value, *a* represents the output value, and *n* represents the total number of samples. The gradient of weight ***W*** and bias ***B*** is derived as follows:

2 $$\frac{\partial C}{{\partial W_{j} }} = \frac{1}{n}\mathop \sum \limits_{x} x_{j} \left( {\sigma \left( z \right) - y} \right)$$

3 $$\frac{\partial C}{{\partial B}} = \frac{1}{n}\mathop \sum \limits_{x} \left( {\sigma \left( z \right) - y} \right)$$

The larger the error and the gradient are, the faster the weight ***W*** and bias ***B*** are adjusted.

### Depthwise separable convolution

Depthwise separable convolution is a special convolution method that operates on space and depth. The core concept is to decompose a complete convolution operation into two steps, namely depthwise convolution and pointwise convolution^22^. The depthwise convolution applies a convolution kernel to each input channel for filtering. The pointwise convolution applies a 1 × 1 convolution to combine the outputs of the depthwise convolution. This decomposing has the effect of drastically reducing computation and model size.

#### Depthwise convolution

**Figure 2:**
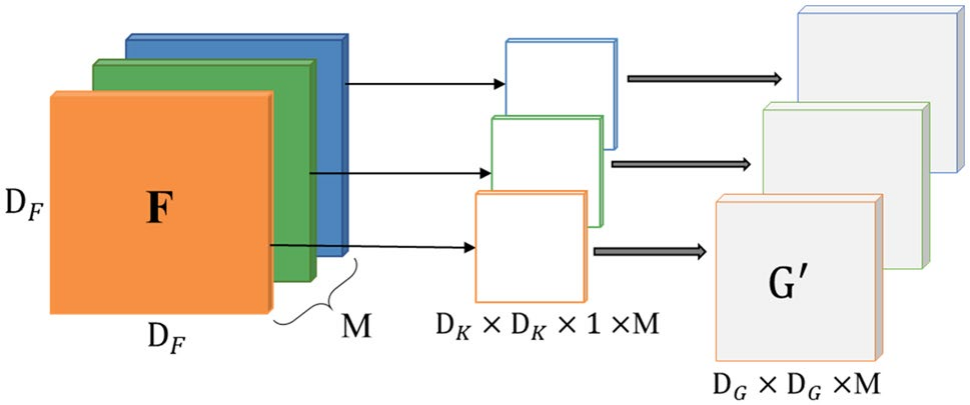
Depthwise convolution.

Depthwise convolution applies a single convolution kernel to each input channel, and outputs corresponding feature maps. Assuming the input feature map **F** is (D_*F*_, D_*F*_, M), the output feature map **G** is (D_*G*_, D_*G*_, N), and the standard convolution kernel **K** is (D_*K*_, D_*K*_, M, N), M and N indicate the number of input and output channels separately. D represents the size of the feature map. **K** is split into a depthwise convolution (D_*K*_, D_*K*_, 1, M) and a pointwise convolution (1, 1, M, N). The depthwise convolution process is shown in Fig. 2.

#### Pointwise convolution

**Figure 3:**
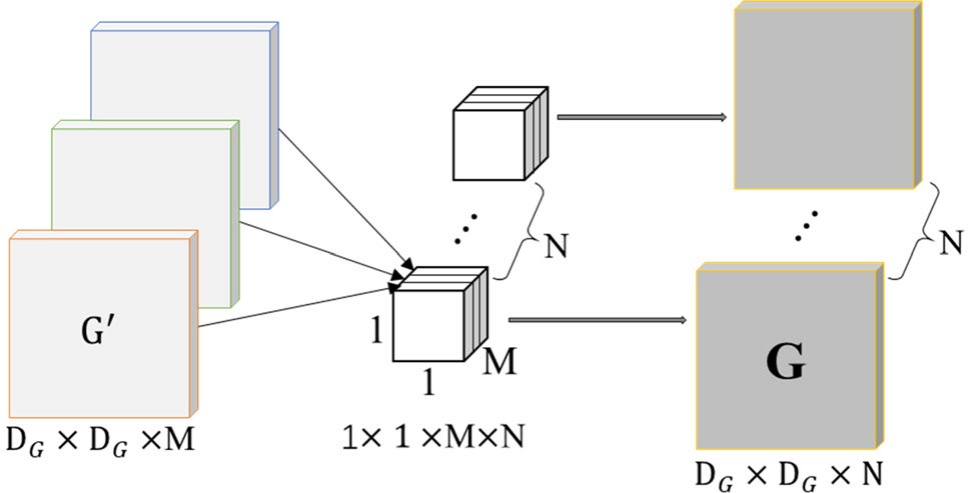
Pointwise convolution.

Pointwise convolution uses the conventional 1 × 1 convolution kernel, and projects the channel calculated by the depthwise convolution onto the new channel space. After depthwise convolution, the pointwise convolution uses N convolution kernels sized 1 × 1 × M to convolve the M D_*G*_ × D_*G*_ feature maps, and then perform weighted combination in the depth direction to output N D_*G*_ × D_*G*_ × 1 feature maps **G** (D_*G*_, D_*G*_, N). The Pointwise Convolution process is shown in Fig. 3, where the number of convolution kernels determines the number of feature maps.

#### Efficiency analysis

The convolution uses a convolution kernel to filter and combine the features to produce a new representation. The depthwise separable convolution decomposes filtering and combination into two steps, thereby breaking the interaction between the output channels and the kernels, and the quantity of the channels and the kernel sizes are greatly reduced the computational cost^24^.

For the input image **F** (D_*F*_, D_*F*_, M), the standard convolution kernel **K** (D_*K*_, D_*K*_, M, N). The formula of the standard convolution is:

4 $${\mathbf{G}}_{k,l,n} = \mathop \sum \limits_{i,j,m} {\mathbf{K}}_{i,j,m,n} \times {\mathbf{F}}_{k + i - 1,l + j - 1,m}$$

The computational cost of the standard convolution is:

5 $${\text{D}}_{K} \times {\text{D}}_{K} \times {\text{M}} \times {\text{N}} \times {\text{D}}_{F} \times {\text{D}}_{F}$$

The formula of the depthwise separable convolution is:

6 $${\hat{\mathbf{G}}}_{k,l,m} = \mathop \sum \limits_{i,j} {\hat{\mathbf{K}}}_{i,j,m} \times {\mathbf{F}}_{k + i - 1,l + j - 1,m}$$

The computational cost of the depthwise separable convolution is:

7 $${\text{D}}_{K} \times {\text{D}}_{K} \times {\text{M}} \times {\text{D}}_{F} \times {\text{D}}_{F} + {\text{M}} \times {\text{N}} \times {\text{D}}_{F} \times {\text{D}}_{F}$$

To illustrate how the proposed model has high computation efficiency, we define a ratio of calculation consumption (RCC) to express it:

8 $$RCC = \frac{{{\text{D}}_{K} \times {\text{D}}_{K} \times {\text{M}} \times {\text{D}}_{F} \times {\text{D}}_{F} + {\text{M}} \times {\text{N}} \times {\text{D}}_{F} \times {\text{D}}_{F} }}{{{\text{D}}_{K} \times {\text{D}}_{K} \times {\text{M}} \times {\text{N}} \times {\text{D}}_{F} \times {\text{D}}_{F} }} = \frac{1}{N} + \frac{1}{{{\text{D}}_{K}^{2} }}$$

From Eq. (5), it is easy to find that the RCC is far smaller than one and proximity to one of N. This indicates that with the map size increasing, the proposed method has lower cost and higher efficiency. The explanation for the efficiency improvement is that the model can learn apart features information of the spaces and the channels when executing the depthwise separable convolution. The depthwise separable convolution makes the detection speed faster and the size of model smaller by reducing the number of calculation parameters.

### Multi-scale gated multi-head attention mechanism (MGMA)

In the CNN model, the pooling layer and the convolution layer are often applied together to reduce the dimension of the input features and the calculation cost. However, too many pooling layers result in the loss of information about small targets in the deep feature maps. In this paper, a multi-scale gated multi-head attention mechanism (MGMA) is proposed to avoid the drawback.

The attention mechanism^23^ is a signal processing mechanism of human vision. It makes full use of limited visual resources to focus on specific vision areas selectively. The attention mechanism has been applied in deep learning recently and widely used in image processing^25^, speech recognition^26^, and natural language processing^27^, and so on. The core task of attention mechanism is to select critical information from mass information quickly and accurately. Compared with the standard convolution calculation, the attention mechanism is characterized by high accuracy, fewer parameters and lower calculation cost.

#### Scaled dot-product attention

The essence of the attention mechanism is a process of weighted summation of ‘Value’ based on ‘Query’ and ‘Key’, and redistribution of weights. From the formal point of view, the attention mechanism can be understood as a key-value query, which maps queries and key-values to the output. The output is the weighted summation of ‘Value’, and the weights are the similarities of ‘Query’ and ‘Key’^28^.

**Figure 4:**
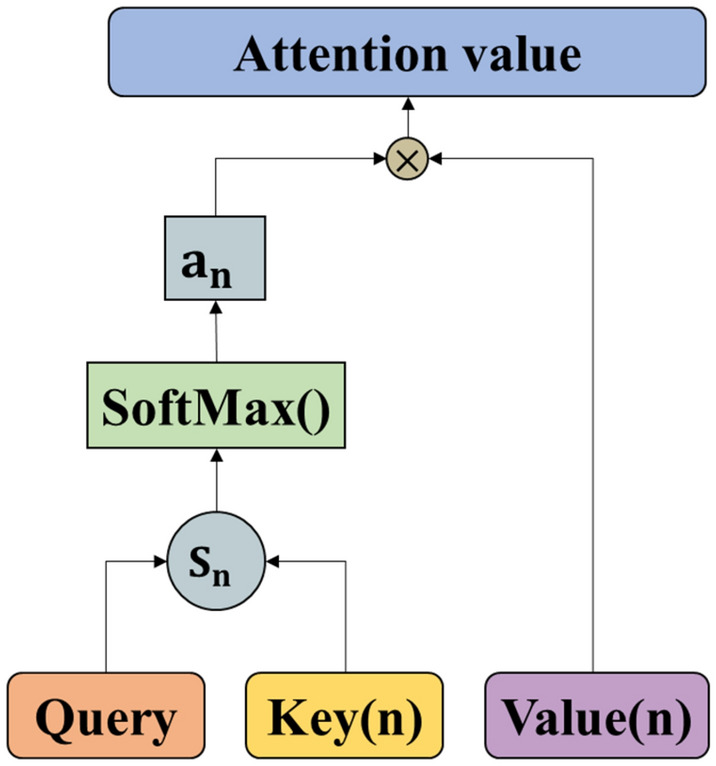
Attention calculation process.

As shown in Fig. 4, the ‘Key’ dimension d_*k*_ and the ‘Value’ dimension d_*v*_ are input into the network. The similarity between ‘Query’ and ‘Key’ is calculated by dot product operation and divided by $\sqrt {{\text{d}}_{k} }$. The weights of each ‘Value’ through the softmax function are obtained.

In practice, the ‘Query’, ‘Key’, and ‘Value’ are packaged into matrices ${\mathbf{Q}},\;{\mathbf{K}}$ and ${\mathbf{V}}$, which are used in the same set of queries for the operation of the attention function. The similarity between the ‘Query’ and multiple ‘Keys’ is calculated when a certain ‘Query’ is given, and the weight coefficient of each ‘Key’ is obtained corresponding to each ‘Value’. The dot product is considered to carry out as Eq. (6):

9 $${\text{S}}_{i} \left( {{\varvec{Q}},{\varvec{K}}_{i} } \right) = \frac{{Q \times K_{i}^{T} }}{{\sqrt {d_{k} } }}$$

Here, $\frac{1}{{\sqrt {d_{k} } }}$ is a scale factor in the formula, which is mainly used to adjust the calculation result. The numerical conversion of the original score can be completed and normalized through a classifier softmax, by which the original calculated score can be sorted into a probability distribution with the sum of all element weights. Meanwhile, it can set the weights for more important elements through the internal mechanism of the softmax classifier. The following formula is used for the output ${\text{a}}_{i}$ of softmax function.

10 $${\text{a}}_{i} = {\text{Softmax}}\left( {{\text{S}}_{i} } \right) = \frac{{exp^{{{\text{S}}_{i} }} }}{{\mathop \sum \nolimits_{i = 1}^{{\text{n}}} exp^{{{\text{S}}_{i} }} }}$$

The scaled dot-product attention can be expressed as:

11 $${\text{Attention}}\left( {{\varvec{Q}},{\varvec{K}}_{i} ,{\varvec{V}}_{i} } \right) = {\text{Softmax}}\left( {\frac{{Q \times K_{i}^{T} }}{{\sqrt {d_{k} } }}} \right) \times V_{i}$$

The whole process of attention mechanism calculation is firstly to carry out dot product similarity calculation of ‘Query’ and ‘Key’, then to obtain the weight coefficient by used softmax function, and finally to carry out the weighted summation of ‘Value’ according to the weight coefficient.

#### Multi-head attention mechanism

**Figure 5:**
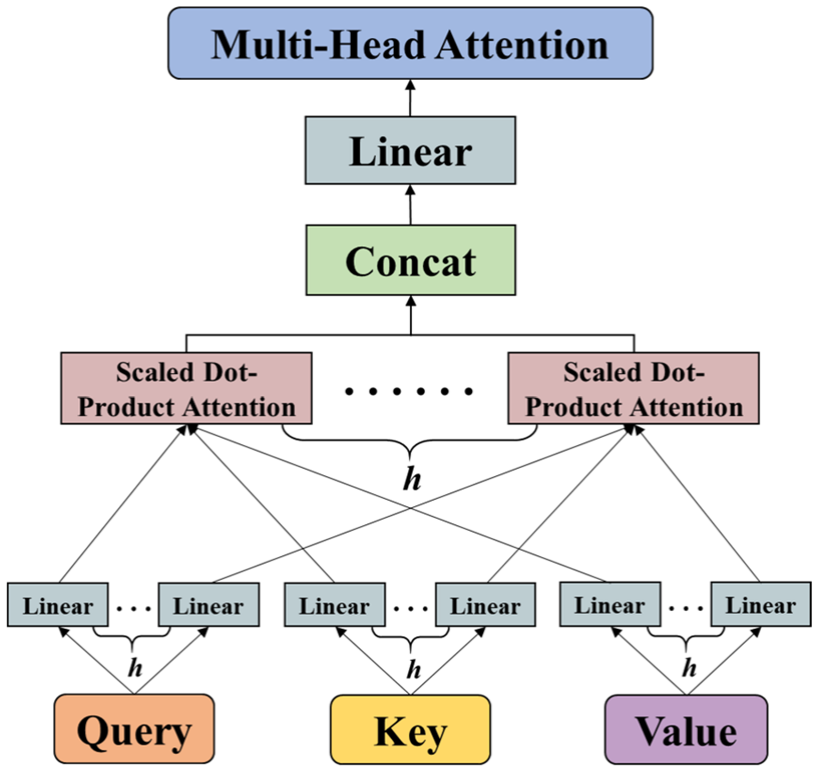
Multi-head attention mechanism.

The multi-head attention mechanism is a special scaled dot-product attention calculation approach. As shown in Fig. 5, the multi-head attention mechanism learns a variety of mappers through the model. First, the linear transformation is carried out on each d_*model*_ of ‘Query’, ‘Key’, and ‘Value’, and then it calculates its scaled dot product attention in parallel to generate a d_*v*_-dimensional output. The multiple (ℎ times) outputs are integrated by Concat function, and linear transformation is performed again to obtain the final output value.

The multi-head attention mechanism does not increase the complexity of the algorithm, but enables the model to learn the correlation information in the different representation subspaces, and improves the model’s perception ability. The calculation formula is shown as follows:

12 $$\begin{aligned} & {\text{Multihead}}\left( {{\varvec{Q}},{\varvec{K}},{\varvec{V}}} \right) = {\text{Concat}}\left( {{\text{head}}_{1} , \ldots ,{\text{head}}_{{\text{h}}} } \right)W^{O} \\ & {\text{head}}_{{\text{i}}} = {\text{Attention}}\left( {QW_{i}^{Q} ,KW_{i}^{K} ,VW_{i}^{V} } \right),W_{i}^{Q} \in R^{{d_{model} \times d_{k} }} ,W_{i}^{K} \in R^{{d_{model} \times d_{k} }} , W_{i}^{V} \in R^{{d_{model} \times d_{v} }} ,W^{O} \in R^{{d_{model} \times hd_{v} }} , \\ \end{aligned}$$

In this paper, there are 8 parallel attention layers, which indicates *h* = 8. Set d_*k*_ = d_*v*_ = d_*model*_*/h* = 64. Due to the dimensionality of each attention layer decreases, the total computational cost is similar to that of the full-dimensional single-head attention layer. Compared with the scaled dot-product attention mechanism, the multi-head attention mechanism has lower complexity and allows the model to learn different representation information avoiding the loss of small target information due to the average value.

#### Multi-scale gated multi-head attention

The multi-scale gated multi-head attention (MGMA) model integrates the multi-size feature maps at different scales by introducing additional branches, and aggregates the global characteristics of multiple dimensions to the output. It avoids information loss of small and medium-sized objects in deep feature maps without additional convolution and pooling calculation, thus enhancing the detection ability of the model to small proportion objects.

**Figure 6:**
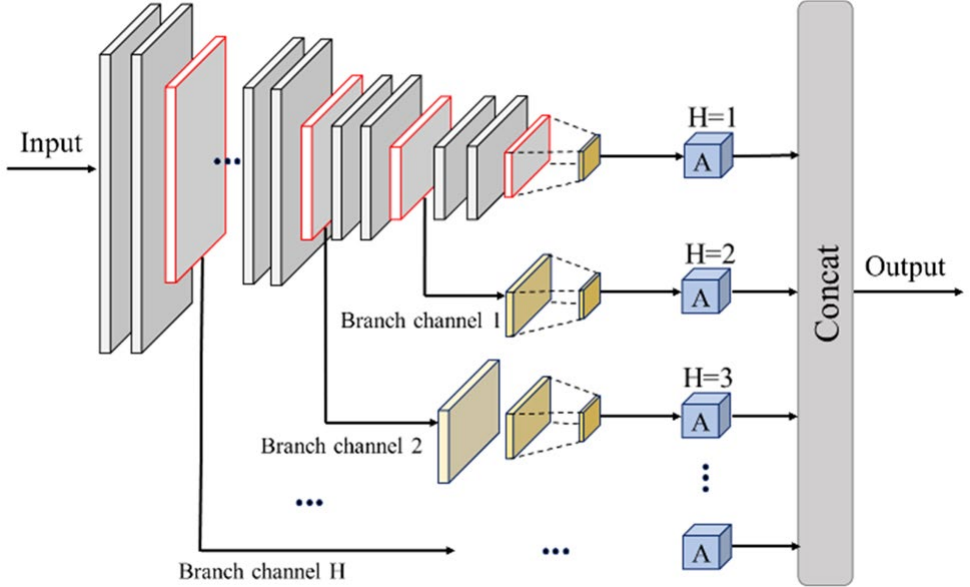
The diagram of MGMA structural.

MGMA transmits the changed feature images to each independent branch channel, and calculates the multi-head attention mechanism of each scale channel in the feature maps. Then, the weighted output of feature maps by multiple scale channels is integrated to obtain the multi-scale feature information. The structure is shown in Fig. 6.

In Fig. 6, the ‘H’ represents the number of branches of the gated attention channel. ‘A’ represents the multi-head attention computing layer. The number of multi-head attention computing layers is the same as the number of channel branches H. In MGMA, when the size of convolutional neural network changes (usually after the maximum pooling layer), the feature information of this layer will be input into the 1 × 1 layer for dimensional transformation, then input into MGMA to extract the features.

In MGMADS-Net, the deep separable convolution can effectively reduce the computational parameters of the model. In order to further improve the classification accuracy of the model, MGMA uses a multi-head attention mechanism to integrate the results of attention operations in different subspaces of multiple branching channels. As a result, MGMADS-Net model has improved the attention to targets of different sizes, especially small and medium-sized targets in large-size images, thus improves the classification accuracy of the model.

## Dataset preparations

### Data collection

The CT images and X-ray images of the COVID-19 patients are collected as the experimental dataset from GitHub (https://github.com/ieee8023/covid-chestxray-dataset), Kaggle (https://www.kaggle.com/paultimothymooney/chest-xray-pneumonia), Kesci (https://www.kesci.com/mw/dataset/5e746ec998d4a8002d2b0861) and Wuhan Tongji Hospital^29^.

**Figure 7:**
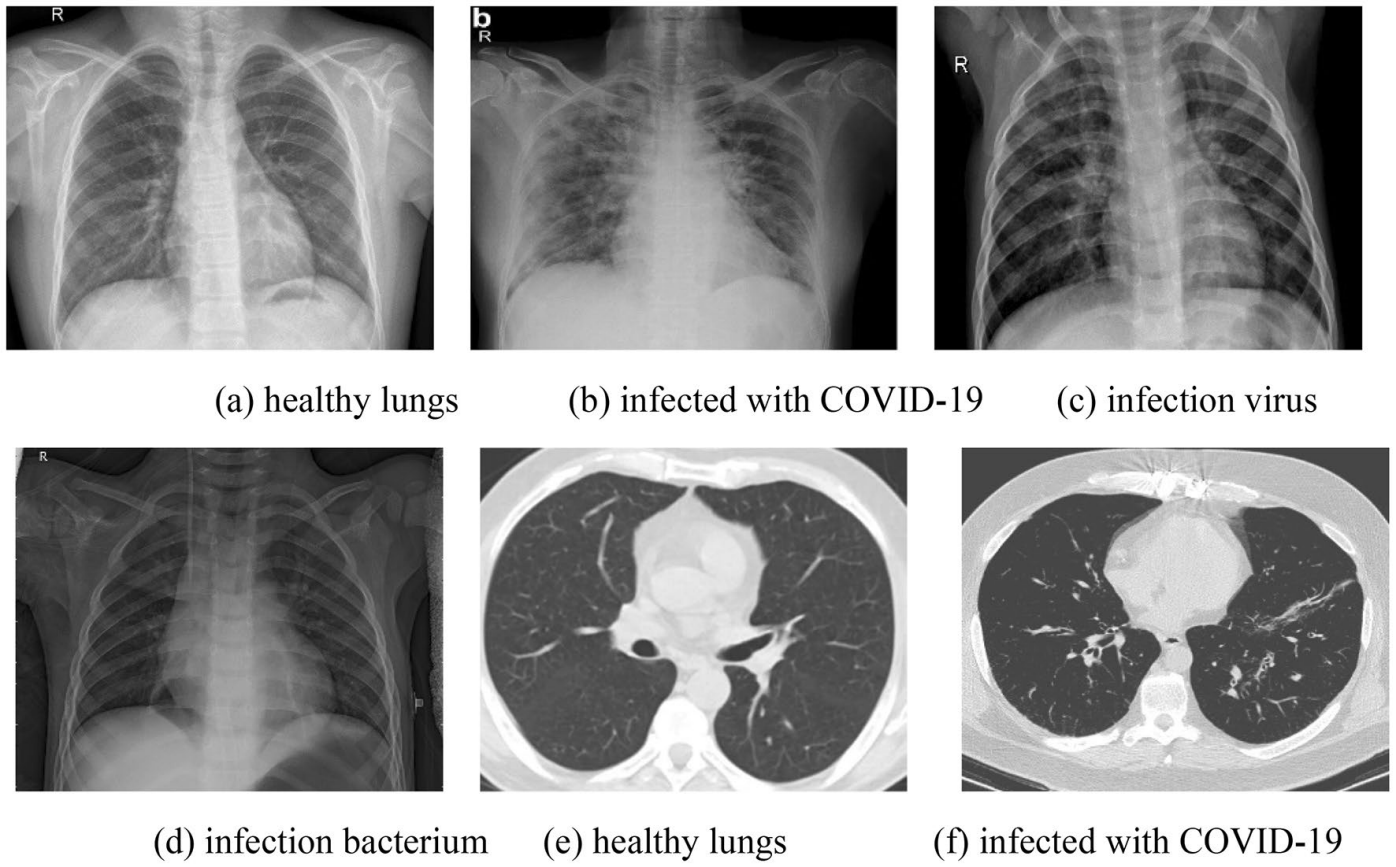
X-ray images (**a**–**d**) and CT images (**e**–**f**).

These datasets are marked by hospital experts with scientific rigor. The image samples are shown in Fig. 7:

According to the dataset distribution, we conduct four-classification on X-ray images and binary classification on CT images as Fig. 7 shown.

### Dataset settings

The distribution of the collected dataset has a data unbalance problem distinctly, which makes the classifier bias towards the class with more samples, which goes against the model's generalization characteristic and the objective judgement of the models.

Data augmentation technique is a way to solve the problems of data shortage and unbalance. It is a popular valid approach to avoid network overfitting when training the model in the current researches. In this paper, the augmentation methods such as affine transformation^20^, image mirroring^30^ and position transformation^31^, are used to expand and enhance the dataset.

#### Affine transformation

Affine transformation includes rotation, translation, scaling, reflection and shearing, which can increase the amount of synthesized data and improve the robustness of the model. The principle of affine transformation can be described as follows.

The image is randomly rotated along the X axis and Y axis, and the enhancement matrix is:

13 $$\left[ {\begin{array}{*{20}c} {x^{\prime}} \\ {y^{\prime}} \\ {z^{\prime}} \\ \end{array} } \right] = {\mathbf{A}} \times {\mathbf{B}} \times {\mathbf{C}} \times {\mathbf{D}} \times \left[ {\begin{array}{*{20}c} x \\ y \\ z \\ \end{array} } \right]$$

where **A** = $$\left[ {\begin{array}{*{20}c} 1 & 0 & 0 \\ 0 & {\cos\uptheta } & { - \sin\uptheta } \\ 0 & {\sin\uptheta } & {\cos\uptheta } \\ \end{array} } \right]$$, **B** = $$\left[ {\begin{array}{*{20}c} {\cos\uptheta } & 0 & {\sin\uptheta } \\ 0 & 1 & 0 \\ { - \sin\uptheta } & 0 & {\cos\uptheta } \\ \end{array} } \right]$$, **C** = $$\left[ {\begin{array}{*{20}c} 1 & 0 & {{\upgamma }_{{\text{x}}} } \\ 0 & 1 & {{\upgamma }_{{\text{y}}} } \\ 0 & 0 & 1 \\ \end{array} } \right]$$, **D** = $$\left[ {\begin{array}{*{20}c} {\upmu } & 0 & 0 \\ 0 & {\upmu } & 0 \\ 0 & 0 & {\upmu } \\ \end{array} } \right]$$.

${\uptheta }$ is the rotation transformation angle, ${\upgamma }$ is the shearing factor, ${\upmu }$ is the scaling factor. The matrix ${\mathbf{A}}{\text{ and }}{\mathbf{B}}$ are the rotation transformation matrix, ${\mathbf{C}}$ is the shearing transformation matrix, and ${\mathbf{D}}$ is the random scaling matrix. The pixel coordinate $\left( {{\text{x}},{\text{ y}},{\text{ z}}} \right)$ is converted to ($x^{\prime},{\text{y}}^{\prime},z^{\prime}$).

#### Image mirroring

The horizontal and vertical mirroring transformation are carried out to the raw dataset. The horizontal mirroring takes the vertical center line of the image as the axis, swapping the pixels, that is, swapping the left half and right half of the image. The vertical mirroring takes the horizontal center line of the image as the axis, and reverses the upper half of the image with the off-duty part.

Suppose the image has width and height. Let the width of the image be *width* and the length be *height*. (*x*_0_,*y*_0_) are the coordinates of the original image, (*x*_1_, *y*_1_) are the transformed coordinates:

The horizontal and vertical mirroring:

14 $$\left[ {\begin{array}{*{20}c} {x_{1} } \\ {y_{1} } \\ 1 \\ \end{array} } \right] = \left[ {\begin{array}{*{20}c} { - 1} & 0 & {width} \\ 0 & 1 & 0 \\ 0 & 0 & 1 \\ \end{array} } \right]\left[ {\begin{array}{*{20}c} {x_{0} } \\ {y_{0} } \\ 1 \\ \end{array} } \right],\quad \left[ {\begin{array}{*{20}c} {x_{1} } \\ {y_{1} } \\ 1 \\ \end{array} } \right] = \left[ {\begin{array}{*{20}c} 1 & 0 & 0 \\ 0 & { - 1} & {height} \\ 0 & 0 & 1 \\ \end{array} } \right]\left[ {\begin{array}{*{20}c} {x_{0} } \\ {y_{0} } \\ 1 \\ \end{array} } \right]$$

After data augmentation through above techniques, the datasets used in this paper are shown in Table 2.

**Table 2:** The dataset distribution before and after augmentation.

Group	Category	Before	After	Category	Before	After
Four classes (total: 17,439)	Normal_X	2916	4832	Virus_X	1733	4166
	Bacteria_X	2705	4418	Covid-19_X	1341	4023
Binary classes (total: 10,839)	Normal_CT	2230	5683	Covid-19_CT	1852	5156

In Table 2, there are 17,439 X-ray images including 4832 normal, 4418 bacterial pneumonia, 4166 viral pneumonia and 4023 with COVID-19 X-ray images. The lung CT images include 5683 normal and 5156 with COVID-19. The augmented dataset has more training data which can improve the generalization and the reliability abilities of the model. It is significant to enhance the robustness of the model and overcome the imbalance problem of positive and negative samples.

## Experimental result and analysis

The training, validation and testing experiments are undertaken on the platform of Intel Core i5-9400F with Windows10 64-bit OS and NVIDIA GeForce GTX 1660 GPU. Python 3.6 is used to code the model, and deep learning frameworks such as TensorFlow GPU 1.8.0, CUDA 9.0 and Keras 2.1.4 are used to build the model structure. In addition, the models use the Pycharm 2017 IDE tool and packages such as Numpy, Scikit-Learn, Matplotlib, and Pandas. The proposed MGMADS-CNN backbone network is available publicly for open access at https://github.com/runningreader/MGMADS.

### Performance metrics

There are three targets are taken to evaluate the performance of the coronavirus identification model used in this article. They are specificity (TNR), sensitivity (TPR) and accuracy (ACC) indicators, which are defined as follows:

15 $$TNR = \frac{TN}{{TN + FP,}}\quad TPR = \frac{TP}{{TP + FN, }} \quad FPR = \frac{FP}{{FP + TN,}} \quad ACC = \frac{TP + TN}{{TP + TN + FP + FN}},$$

Here *TP*, *TN*, *FP*, and *FN* are the numbers of true positives, true negatives, false positives, and false negatives, respectively. Generally speaking, high specificity means low misdiagnosis rate, and high sensitivity means low missed diagnosis rate. The higher the accuracy, the better the classification effect.

### Tenfold cross-validation

In order to effectively reduce the variability of test results, this paper adopts k-fold cross-validation to conduct experiments, and randomly divide the dataset D into *k* uniform and disjoint subsets D_1_, D_2_ ,…, D_*k*_, such that:

16 $$\bigcup\limits_{i = 1}^{k} {D_{i} = D_{data} } ,\quad D_{i} \bigcap {D_{j} = \emptyset } ,\quad \forall 1 \le i \ne j \le k,\quad \left| {D_{i} } \right| \approx \frac{{|D_{data} |}}{k},\quad \forall 1 \le i \le k$$

In this paper, *k* = 10. The validation process can be executed as following steps:

*Step 1* The dataset is randomly divided into ten equal parts.

*Step 2* Taking one part as the test set, one as the validation set, and the other eight parts as the training set.

*Step 3* Traning, validating and testing.

*Step 4* Changing the data distribution randomly and execute the Step 2 ten times.

**Figure 8:**
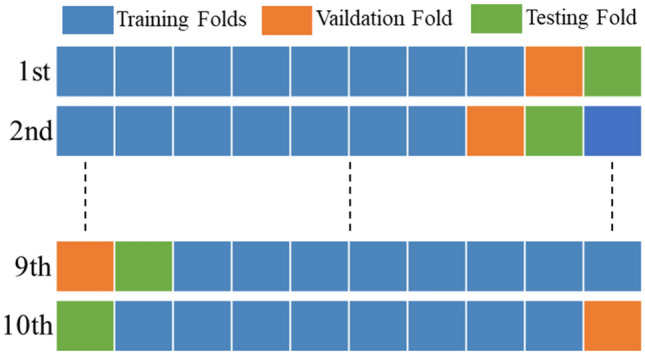
Graphical representation of tenfold cross-validation.

The dataset distribution at each cyclic execution is as shown in Fig. 8.After ten executions, the average accuracy is used to evaluate the performance, and the formula is as follows:

17 $$ACC_{test} = \frac{1}{k}\mathop \sum \limits_{i = 1}^{k} ACC_{i} ,$$

### Training and validation comparisons

**Figure 9:**
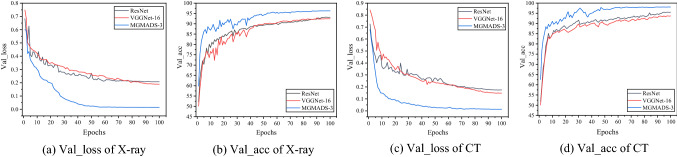
Val_loss and Val_acc curves of X-ray and CT images.

The eight models (LeNet-5, AlexNet, GoogLeNet, ResNet, VGGNet-16, MGMADS-1, MGMADS-2 and MGMADS-3) are conducted on the declared platform and framework. In validation, with each epoch execution, it gets a loss value. After 100 epochs, 100 loss values can be obtained. In order to present the differences among the models directly and graphically, it picks up validation_loss (Val_loss) and validation_accuracy (Val_acc) of ResNet, VGGNet-16 and MGMADS-3 as representatives shown in Fig. 9.

At epoch 100, the three models have Val_loss values of 0.2069, 0.1891 and 0.0140, and the Val_acc values are 93.19%, 92.40% and 96.25% respectively on the X-ray images dataset. Meanwhile, on the CT images dataset, the Val_loss values are 0.1746, 0.1483 and 0.0136 corresponding to the three models, and the Val_acc values are 95.53%, 93.75% and 98.09%. On both X-ray dataset and CT images dataset, the Val_loss and Val_acc of the MGMADS-3 achieve outstanding results. In the view of accuracy, the MGMADS-3 model has achieved a higher value, and the growth trend and oscillation amplitude of the Val_acc curve behave better than that of the ResNet and VGGNet-16 models. It is verified the validity and feasibility of the MGMADS-3 model.

The detailed comparisons on LeNet-5, AlexNet, GoogLeNet, ResNet, VGGNet, MGMADS-1, MGMADS-2 and MGMADS-3 models are list out as Column 3–6 in Table 3. For the four classification of the X-ray images in the Table 2, the Train_loss values (0.0602, 0.0485, 0.0139) and Val_loss values (0.0602, 0.0485, 0.0140) of the MGMADS-CNN models are all far lower than the other five models. With the attention branches H increasing, the loss value goes smaller. It indicates that the proposed model achieves expected effect. The Train_acc values (98.63%, 99.03%, 99.62%) and Val_acc values (93.31%, 94.87%, 96.25%) of the MGMADS-CNN models are all higher than the other five models. Obviously, with the H increasing, the accuracy goes higher.

**Table 3:** Training and validation experiment results@epoch = 100.

Datasets	Methods	Train_loss	Train_acc (%)	Val_loss	Val_acc (%)	Size (M)′	Specificity (%)	Sensitivity (%)	Test_acc (%)
Four classes (X-ray images)	LeNet-5	0.2418	94.72	0.3494	90.72	51.5	89.06	91.27	91.27
	AlexNet	0.2065	95.44	0.3059	90.08	444	92.13	90.13	90.15
	GoogLeNet	0.2832	92.40	0.2944	86.12	52	87.64	89.25	88.12
	ResNet	0.1931	97.15	0.2069	93.19	270	96.73	91.38	93.38
	VGGNet-16	0.1845	97.89	0.1891	92.40	114	94.52	92.05	92.45
	MGMADS-1	**0.0602**	**98.63**	**0.0602**	**93.31**	**40.1**	**96.03**	**93.12**	**93.20**
	MGMADS-2	**0.0485**	**99.03**	**0.0485**	**94.87**	**41.8**	**96.12**	**95.12**	**95.12**
	MGMADS-3	**0.0139**	**99.62**	**0.0140**	**96.25**	**43.6**	**98.06**	**96.60**	**96.75**
Binary classes (CT images)	LeNet-5	0.2044	97.42	0.2244	93.20	51.2	92.76	92.45	92.42
	AlexNet	0.1761	97.39	0.1815	93.75	443	94.17	93.20	93.75
	GoogLeNet	0.1911	95.43	0.1962	92.68	52	90.13	92.30	92.34
	ResNet	0.1483	98.97	0.1746	95.53	270	98.02	95.38	95.85
	VGGNet-16	0.1267	99.09	0.1483	93.75	114	96.81	94.10	94.35
	MGMADS-1	**0.0202**	**99.44**	**0.0367**	**96.37**	**40.1**	**96.52**	**96.34**	**96.34**
	MGMADS-2	**0.0122**	**99.62**	**0.0211**	**97.50**	**41.8**	**97.47**	**97.52**	**97.56**
	MGMADS-3	**0.0025**	**99.93**	**0.0136**	**98.09**	**43.6**	**98.17**	**98.05**	**98.25**

Bold values indicate better results than other values.

For the binary classification of the CT images in the Table 2, the performances of the proposed MGMADS-CNN models are better than that of the X-ray images. For instance, the loss values of the MGMADS-CNN are 0.0202, 0.0122, 0.0025 in training and 0.0367, 0.0211, 0.0136 in validation. The accuracy values are 99.44%, 99.62%, 99.93% in training and 96.37%, 97.50%, 98.09% in validation. The performances of the proposed MGMADS-CNN models do better than the compared typical models.

It is easy to figure out from the Table 3 that no matter the training or the validation, the MGMADS-3 model achieves the highest accuracy than the compared typical models, which has a good recognition effect on X-ray and CT images.

### MGMADS-CNN performances tests

The performances adopted to evaluation the models include the model size, detection speed, the specificity, the sensitivity, and the test accuracy. Another vital evaluation target named receiver operating characteristics (ROC) is presented in the following. At the end, the comparisons with the related published literatures are list out.

#### Test performance

The comparison experimental results are shown Column 7–10 in Table 3 of the eight models as above.

From Table 3, the model sizes of MGMADS-CNN are about 40 M bytes, which much smaller than other networks, resulting in a lighter weighted network structure. With the shrink models, the performances are kept stable and even improved slightly. In terms of model classification, MGMADS-CNN models have higher specificity, sensitivity and accuracy compared with other models. For example, the MGMADS-3 model achieves 98.06%, 96.60% and 96.75% on the X-ray images data, and 98.17%, 98.05% and 98.25% on the CT images data.

**Figure 10:**
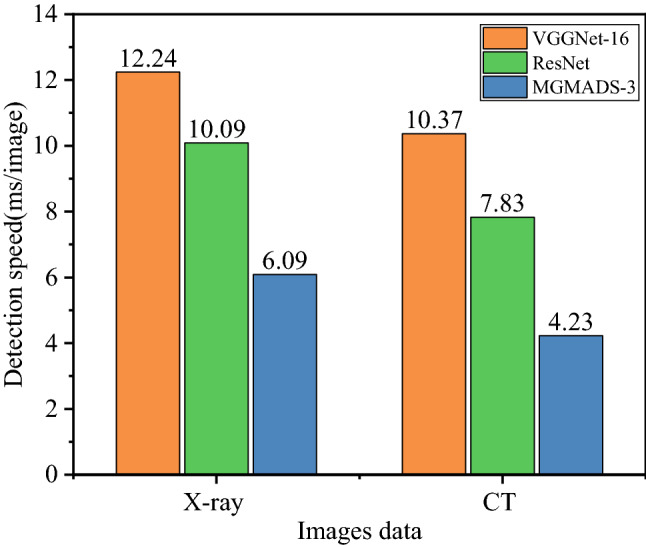
The detection speed comparisons of the three models.

In order to compare the detection speed of the model more intuitively, ResNet and VGGNet-16 models are selected as the comparison networks. The detection speed histograms of ResNet, VGGNet-16 and MGMADS-3 models in X-ray image data and CT image data are shown in Fig. 10.

As shown in Fig. 10, on the X-ray images data, the detection speeds for each image of ResNet, VGGNet-16 and MGMADS-3 model are 12.24 ms, 10.09 ms, 6.09 ms, and on the CT images data, the detection speeds are 10.37 ms, 7.83 ms, 4.23 ms. It can be seen obviously that the detection speeds are improved to a new level either on X-ray images or CT images. It is further verified that the proposed model can detect and classify COVID-19 faster.

#### Receiver operating characteristic curves

**Figure 11:**
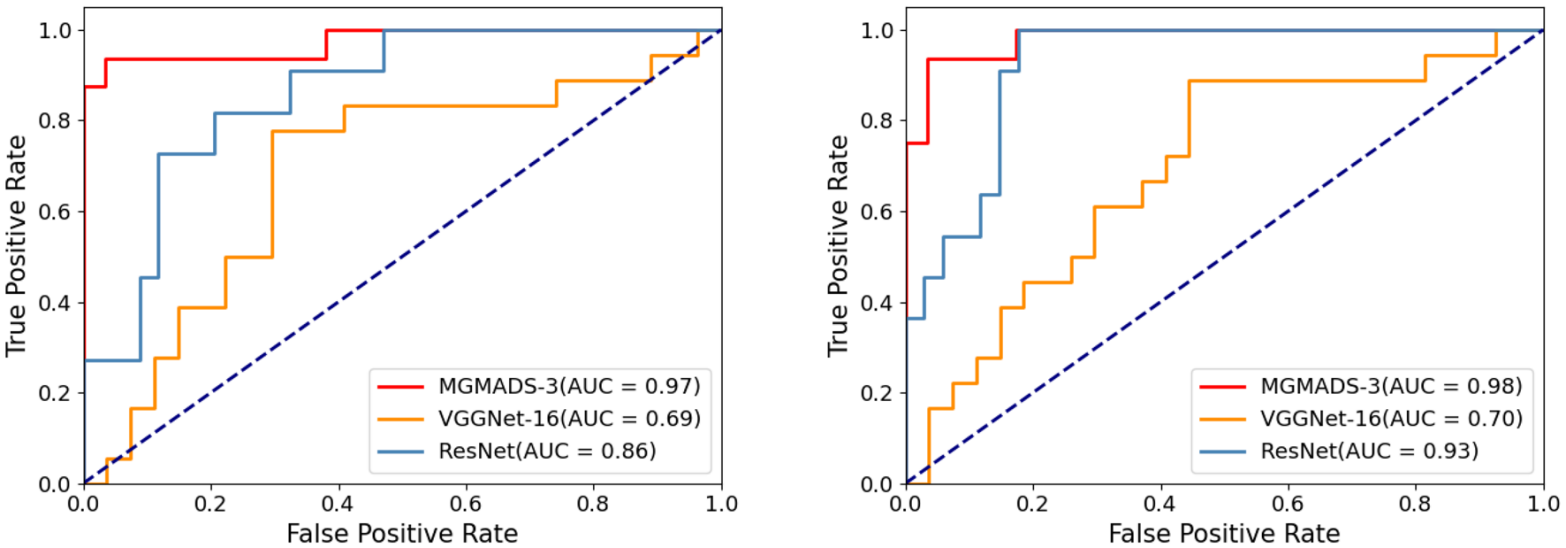
ROC analysis curves of different models in X-ray and CT images.

The Receiver Operating Characteristics (ROC) curve is the plot of True Positive Rate (TPR) against False Positive Rate (FPR). It represents the diagnostic ability of the model by measuring the degree of separability among different classes. The ROC curves on the X-ray images data and CT images data are shown in Fig. 11.

The higher the area under the curve (AUC), the better is the model in distinguishing among different classes. From Fig. 11, the AUC of ResNet, VGGNet-16 and MGMADS-3 are 0.86, 0.69, and 0.97 on X-ray images data, and 0.93, 0.70, and 0.98 on CT images data.

#### Comparison to the related literatures

Considering the related reports published in 2020, the comparisons between the literatures mentioned in the Section ‘Introduction’ and our proposed models are summarized in Table 4. The number in the bracket of the third column represents 2, 3, and 4 classifications.

**Table 4:** Model performance comparison.

Literatures	Methods	Images (classes)	Dataset Quant	Test_acc (%)
Hemdan^13^	COVIDX-Net	X-ray(2)	50	91
Abbas^14^	DeTrac	X-ray(2)	1764	95.12
Ozturk^15^	DarkCovidNet	X-ray(3)	1419	87.02
Khan^16^	CoroNet	X-ray(4)	1300	89.6
Wang^17^	COVID-Net	X-ray(4)	13,975	83.5
**Proposed**	**MGMADS-3**	**X-ray(4)**	**17,439**	**96.75**
Li^19^	COVNet	CT(2)	4356	90
Zheng^20^	DeCovNet	CT(2)	499	90.1
Song^21^	DRE-Net	CT(2)	1485	94.0
**Proposed**	**MGMADS-3**	**CT(2)**	**10,839**	**98.25**

Bold values indicate better results than other values.

As shown in the Table 4, the MGMADS-3 network model proposed in this paper has achieved better classification effects than the other models whether of four-classification on X-ray images or binary classification on CT images.

There are two reasons for the better classification effect of the proposed model. First, the MGMA can effectively extract more features information from different subspaces and learn the correlation information of small targets. Second, the depthwise separable convolution substituting the standard convolution can reduce the number of model parameters. Compared with the typical convolutional structures, the proposed MGMADS-CNN model with lighter structure is featured as faster detection speed, higher classification accuracy, and better efficiency.

## Conclusion

In the paper, a new CNN model named MGMADS-CNN is proposed, which is based on multi-head attention mechanism and depthwise separable convolution. The MGMADS-CNN model has the advantages of small size, fast detection speed and high accuracy. The MGMADS-CNN model can extract small target information from different subspaces by the multi-head attention mechanism, thus improve the accuracy of the model. Compared with the standard convolution, the depthwise separable convolution reduces the number of model calculation parameters, thus reduces the size of the model and improving the detection speed of the model. The model proposed in this paper not only improves the practicability of COVID-19 classification, but also provides a novel idea for computer-aided diagnosis (CAD).

## References

[1] WHO. *Statement on the Second Meeting of the International Health Regulations (2005) Emergency Committee Regarding the Outbreak of Novel Coronavirus (2019-nCoV)* (2020).

[2] World Health Organization, Novel Coronavirus(2019-nCoV) Situation Report-30. https://www.who.int/docs/default-source/coronaviruse/situationreports/20200219-sitrep-30-covid-19.pdf?sfvrsn=6e50645_2. Accessed 24 March 2020.

[3] WHO. Coronavirus disease (COVID-2019) situation reports[R/OL].[2021-6-28]. https://www.who.int/emergencies/diseases/novelcoronavirus-2019/situation-reports/.

[4] Huang C, Wang Y, Li X (2020). Clinical features of patients infected with 2019 novel coronavirus in Wuhan, China. Lancet.

[5] Chung M, Bernheim A, Mei X (2020). CT imaging features of 2019 novel coronavirus (2019-nCoV). Radiology.

[6] Guan W, Ni Z, Hu Y (2020). Clinical characteristics of coronavirus disease 2019 in China. N. Engl. J. Med..

[7] Song F, Shi N, Shan F (2020). Emerging 2019 novel coronavirus (2019-nCoV) pneumonia. Radiology.

[8] Zarifian A, Nour MG, Rezayat AA (2020). Chest CT findings of coronavirus disease 2019 (COVID-19): A comprehensive meta-analysis of 9907 confirmed patients. Clin. Imaging.

[9] Bernheim A, Mei X, Huang M (2020). Chest CT findings in coronavirus disease-19 (COVID-19): Relationship to duration of infection. Radiology.

[10] Xie X, Zhong Z, Zhao W (2020). Chest CT for typical coronavirus disease 2019 (COVID-19) pneumonia: relationship to negative RT-PCR testing. Radiology.

[11] Wang, X., Peng, Y., Lu, L., *et al*. Chestx-ray8: Hospital-scale chest X-ray database and benchmarks on weakly-supervised classification and localization of common thorax diseases. In *Proceedings of the IEEE Conference on Computer Vision and Pattern Recognition* 2097–2106 (2017).

[12] Rajpurkar, P., Irvin, J., Zhu, K., *et al*. Chexnet: Radiologist-level pneumonia detection on chest x-rays with deep learning. arXiv preprint arXiv:1711.05225 (2017).

[13] Hemdan, E. E. D., Shouman, M. A. & Karar, M. E. Covidx-net: A framework of deep learning classifiers to diagnose covid-19 in X-ray images. arXiv preprint arXiv:2003.11055 (2020).

[14] Abbas A, Abdelsamea MM, Gaber MM (2021). Classification of COVID-19 in chest X-ray images using DeTraC deep convolutional neural network. Appl. Intell..

[15] Ozturk T, Talo M, Yildirim EA (2020). Automated detection of COVID-19 cases using deep neural networks with X-ray images. Comput. Biol. Med..

[16] Khan AI, Shah JL, Bhat MM (2020). Coronet: A deep neural network for detection and diagnosis of COVID-19 from chest X-ray images. Comput. Methods Prog. Biomed..

[17] Wang L, Lin ZQ, Wong A (2020). Covid-net: A tailored deep convolutional neural network design for detection of covid-19 cases from chest X-ray images. Sci. Rep..

[18] Singh D, Kumar V, Kaur M (2020). Classification of COVID-19 patients from chest CT images using multi-objective differential evolution-based convolutional neural networks. Eur. J. Clin. Microbiol. Infect. Dis..

[19] Li L, Qin L, Xu Z (2020). Using artificial intelligence to detect COVID-19 and community-acquired pneumonia based on pulmonary CT: evaluation of the diagnostic accuracy. Radiology.

[20] Wang X, Deng X, Fu Q (2020). A weakly-supervised framework for COVID-19 classification and lesion localization from chest CT. IEEE Trans Med Imaging.

[21] Song Y, Zheng S, Li L (2021). Deep learning enables accurate diagnosis of novel coronavirus (COVID-19) with CT images. IEEE/ACM Trans. Comput. Biol. Bioinform..

[22] Chollet, F. Xception: Deep learning with depthwise separable convolutions. In *Proceedings of the IEEE Conference on Computer Vision and Pattern Recognition* 1251–1258 (2017).

[23] Mnih, V., Heess, N. & Graves, A. Recurrent models of visual attention. In *Advances in Neural Information Processing Systems* 2204–2212 (2014).

[24] Howard, A. G., Zhu, M., Chen, B., *et al*. Mobilenets: Efficient convolutional neural networks for mobile vision applications. arXiv preprint arXiv:1704.04861 (2017).

[25] Tian C, Yong Xu, Li Z (2020). Attention-guided CNN for image denoising. Neural Netw..

[26] ShanYang HL, Kang S (2020). On the localness modeling for the self-attention based end-to-end speech synthesis. Neural Netw..

[27] Chen X, Wang T, Zhu Y (2020). Adaptive embedding gate for attention-based scene text recognition. Neurocomputing.

[28] Vaswani A, Shazeer N, Parmar N (2017). Attention is all you need. Adv. Neural Inf. Process. Syst..

[29] Zhao, J., Zhang, Y., He, X., *et al*. COVID-CT-dataset: A CT scan dataset about COVID-19. arXiv preprint arXiv:2003.13865 (2020).

[30] Apostolopoulos ID, Aznaouridis SI, Tzani MA (2020). Extracting possibly representative COVID-19 biomarkers from X-ray images with deep learning approach and image data related to pulmonary diseases. J. Med. Biol. Eng..

[31] Farooq, M. & Hafeez, A. Covid-resnet: A deep learning framework for screening of covid19 from radiographs. arXiv preprint arXiv:2003.14395 (2020).

